# Recognizing New Classes with Synthetic Data in the Loop: Application to Traffic Sign Recognition

**DOI:** 10.3390/s20030583

**Published:** 2020-01-21

**Authors:** Gabriel Villalonga, Joost Van de Weijer, Antonio M. López

**Affiliations:** 1 Computer Vision Center (CVC), Universitat Autònoma de Barcelona (UAB), 08193 Bellaterra, Spain; joost@cvc.uab.es (J.V.d.W.); antonio@cvc.uab.es (A.M.L.); 2 Computer Science Department, Universitat Autònoma de Barcelona (UAB), 08193 Bellaterra, Spain

**Keywords:** CNNs, training with synthetic data, traffic sign recognition

## Abstract

On-board vision systems may need to increase the number of classes that can be recognized in a relatively short period. For instance, a traffic sign recognition system may suddenly be required to recognize new signs. Since collecting and annotating samples of such new classes may need more time than we wish, especially for uncommon signs, we propose a method to generate these samples by combining synthetic images and Generative Adversarial Network (GAN) technology. In particular, the GAN is trained on synthetic and real-world samples from known classes to perform synthetic-to-real domain adaptation, but applied to synthetic samples of the new classes. Using the Tsinghua dataset with a synthetic counterpart, SYNTHIA-TS, we have run an extensive set of experiments. The results show that the proposed method is indeed effective, provided that we use a proper Convolutional Neural Network (CNN) to perform the traffic sign recognition (classification) task as well as a proper GAN to transform the synthetic images. Here, a ResNet101-based classifier and domain adaptation based on CycleGAN performed extremely well for a ratio ∼1/4 for new/known classes; even for more challenging ratios such as ∼4/1, the results are also very positive.

## 1. Introduction

On-board computer vision is fundamental to perceiving the traffic environment around the ego-vehicle and, therefore, a crucial technology for assisting drivers or enabling fully autonomous vehicles (AVs). In the last decade, computer vision has been empowered by the use of Convolutional Neural Networks (CNNs), which allow the extraction of very detailed meaningful information from raw images. Over the last few years, we have witnessed unprecedented breakthroughs in tasks such as image-level classification [1,2], bounding-box-level 2D and 3D object detection [3,4,5,6,7], pixel-wise class and instance segmentation [8,9,10,11,12,13,14], skeleton-wise human pose estimation [15,16,17], and even dense monocular depth estimation [18,19,20,21,22,23,24]; all of them, among others, are essential visual capabilities for high levels of driving automation or assistance.

Indeed, CNNs have become very accurate models provided that there is sufficient data (in size and diversity) for their training [25,26]; *data* refers to both the raw images and the ground truth (GT) that we must associate with them as training supervision. In fact, since CNNs are data hungry and most GT comes from a (cumbersome and error-prone) manual labeling process, providing GT at scale, reducing manual intervention, and/or reusing previous knowledge have become emerging topics for computer vision research in general and for autonomous driving in particular. For instance, *active learning* techniques [27,28,29,30] focus on automatically finding the a priori best training images for their posterior manual labeling out of a large number of unlabelled ones; *self-labeling* techniques [31,32,33] focus on progressively self-labeling images by refining increasingly accurate models; *transfer learning* techniques [34,35] focus on reusing existing models for re-training them to perform new tasks, so that only labels for such tasks are required; *domain adaptation* techniques [36,37,38,39] focus on reusing existing models in new domains, minimizing the labeling effort required in the new domains; while *self-supervision* [40,41,42,43] focuses on learning visual models without manual labeling, with the support of auxiliary simple (pretext) tasks for which it is possible to automatically define self-labels. Overall, all of these research topics are evidence that, due to CNNs, computer vision and its applications have become strongly data-dependent.

In this context, situations where a sudden lack of data can seriously limit the operational capabilities of a computer vision system can appear. For instance, imagine an AV or a driver assistance system that must *detect* (i.e., localize within an image) and *recognize/classify* (i.e., assign a specific meaning) traffic signs using on-board computer vision. While detection itself can be very robust due to the stability of the shape of traffic signs across the world (triangles, rectangles, circles), it can happen that the vehicles of a fleet deployed in a new world area suddenly detect traffic signs that they cannot recognize. These vehicles can automatically broadcast the unknown traffic signs to the company for opening an incidence. If the company decides that it is required to actually recognize the new traffic signs, it may take too much time to collect sufficient samples and perform their proper labeling, especially if the samples must be acquired under a variety of environmental conditions and viewpoints, which can be challenging if such new traffic signs are scarce (i.e., they are rare events). While this is just an illustrative example, we can imagine analogous situations for robots manipulating goods, traffic surveillance systems separating vehicles according to their functionality, etc.; readers can imagine other examples where perception-based systems may have such a kind of *unknown class* problem; after all, both CNNs as well as traditional (shallow) vision models work under the *closed-world* assumption.

Following a new tendency of recent years [44,45,46,47,48,49], in this paper, we propose the exploration of how synthetic data can help in such situations. In particular, assuming that we have a few samples of an unknown class (keeping with the example, these can be traffic sign images reported by the fleet), we propose a method consisting of the following steps: (1) To elaborate 3D graphical instances of the class and automatically place them all around in a virtual environment; (2) by varying the simulation conditions within the virtual environment, to automatically generate as many image samples as needed; (3) to domain-adapt these samples to close the visual gap with real-world images; (4) to retrain the corresponding CNNs for recognizing the new class. Then, the research question is how these results would differ from the results obtained by a counterpart procedure consisting of capturing samples of the new class with real-world cameras and labeling them by hand (i.e., instead of the steps (1)–(3)). Note that (3) must be a task-agnostic domain adaptation step. In particular, we propose the use of a Generative Adversarial Network (GAN) [50], previously trained in a generic manner to transform our synthetic images to look like real-world images captured by the cameras of our computer vision system. We remark that such a GAN has never seen samples of the unknown class before. As a very important and challenging use case, we assess the success of our proposal by applying it to traffic sign recognition in the wild [51], which can be seen as a fine-grain image classification task. We will elaborate exhaustive experiments by varying the known and unknown traffic signs according to different criteria, not only for training/retraining the classification CNNs, but also the task-agnostic GAN. In order to support this experimental part, we have created a synthetic dataset of traffic signs using an evolution of our SYNTHIA environment [44], which we call SYNTHIA-TS. Using the Tsinghua dataset [51] and SYNTHIA-TS, we ran an extensive set of experiments which show that the proposed method is indeed effective, provided that we use a proper CNN to perform the traffic sign classification task as well as a proper GAN to transform the synthetic images.

Here, a ResNet101-based classifier and a CycleGAN performed extremely well for a ratio of ∼1/4 for new/known classes; even for more challenging ratios such as ∼4/1, the results are also very positive. Therefore, synthesizing data following our proposal establishes a proper methodology to minimize the lack of real-world labeled data when a computer vision system must be retrained to recognize new classes in a relatively short period.

The rest of the paper is organized as follows. Section 2 reviews the most related work to our proposal. Section 3 elaborates our proposal. Section 4 details the experimental protocol, the obtained results, and the conclusions deduced from them. Finally, Section 5 summarizes the work presented in this paper and suggests future directions of research in line with our conclusions.

## 2. Related Work

**Figure 1:**
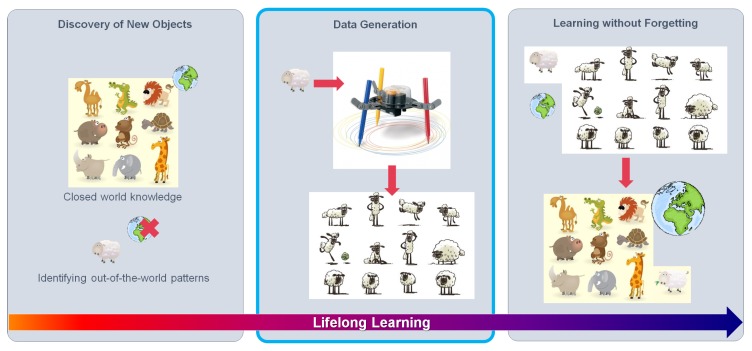
Lifelong learning setting. First, unknown classes are identified. Then, if we want to consider them in the future, we must collect diverse samples of these classes for posterior model retraining. Finally, we retrain the models to recognize the new classes without forgetting previous ones. In this paper, we focus on the second step, assuming that rather than collecting the samples from the real world, we generate them by using a virtual world.

Learning to recognize new classes falls into the paradigm of *lifelong learning* [52,53,54], where a perception-based system has to continuously adapt to situations not previously experienced. We can think of three main components of such a learning capability (see Figure 1). The first one consists of the ability to identify unknown classes, which is not trivial, since CNNs tend to be overconfident about their classification decisions. In fact, this is an active research topic known as *out-of-distribution detection* (which includes *novelty* and *anomaly* detection; this paper relates to the novelty case) [55,56,57,58]. The second component consists of a data generation protocol to collect training samples of these unknown classes, provided that the CNN needs to take them into account in the future. Finally, the third component consists of a procedure that allows the CNN to recognize the new classes without deteriorating its accuracy in identifying the classes for which it was initially/previously trained; this is known as learning without forgetting [59,60,61,62] and still is an open and challenging research topic. In fact, the work of [58] focuses on out-of-distribution for our use case, i.e., traffic sign recognition.

In this paper, we focus on data generation. We require that we are given the unknown (novel) traffic signs in the form of a few on-board captured images. In addition, to avoid the forgetting problem when retraining the traffic sign recognition CNNs, we will just retrain using all of the available data (known and synthesized); in this way, the paper can really focus on assessing the usefulness of synthesizing samples of the unknown classes. Accordingly, the remainder of this section addresses the use of synthesized visual data for training computer vision models, as well as the use of GANs to perform task-agnostic domain adaptation.

Researchers such as Taylor et al. [63] pioneered the use of videogame data for testing vision-based tracking algorithms. Marin et al. [64] extended the use of this synthetic data to train object detectors performing in real images, while Vazquez et al. [65] raised the attention on the domain gap between virtual and real world images. From there, the use of synthetic visual data generated from virtual environments has kept growing. We found works using synthetic data for object detection/recognition [66,67,68,69], object viewpoint recognition [70], re-identification [71], and human pose estimation [72]; building synthetic cities for autonomous driving tasks such as semantic segmentation [44,73], place recognition [74], object tracking [45,75], object detection [76,77], stixel computation [78], and benchmarking different on-board computer vision tasks [47]; building indoor scenes for semantic segmentation [79], as well as normal and depth estimation [80]; generating GT for optical flow, scene flow, and disparity [81,82]; generating augmented reality images to support object detection [83]; simulating adverse atmospheric conditions such as rain or fog [84,85]; even performing procedural generation of videos for human action recognition [86,87]. Moreover, since robotics and autonomous driving rely on sensorimotor models worthy of being trained and tested dynamically, in the last years, the use of simulators has been intensified beyond datasets [48,49,88,89].

In contrast to this literature, we can leverage the already-annotated real-world images conveying a set of classes known by our current CNN-based classifier, but we have to assess the possibility of using automatically generated synthetic images as samples of classes that are unknown for our current CNN. Therefore, these synthetic images must be used to retrain the CNN to properly classify previous and new classes. We will evaluate two different settings: First, when the synthetic images are used as they come from the virtual environment; second, when the synthetic images are transformed by a GAN to look like the real-world images, i.e., as a type of task-agnostic domain adaptation where, following the domain adaptation terminology, the synthetic world acts as the *source* domain and the real world as the *target* domain.

GANs use a *generator* CNN to transform the appearance of source images to look like target images, and a *discriminator* CNN which aims at distinguishing between the transformed and the original images in the target domain. The generator–discriminator system is trained until the discriminator is not able to distinguish the origin of the images, which is understood as the point when the source images are similar enough to the target ones. This application of GANs is known as *image-to-image translation*.

Isola et al. [90] proposed an encoder–decoder as the generator architecture, and a patch-based (patchGAN) approach for the discriminator. Since this approach was only able to work with low-resolution images, other approaches build upon this method to overcome this problem [91]. However, a relevant observation is that these proposals require pixel-level GT about how the generated images should look, which is termed as supervised image-to-image translation. In order to avoid this kind of supervision, Taigman et al. [92] designed an encoder–decoder generator in such a way that the encoder features are indistinguishable for original and transformed images. In other words, for the GT, it is only required to know if the images come from the source domain or the target one, which is always possible at training time. Liu et al. [93] also focused on generators’ feature layers. Afterwards, other alternatives were proposed that did not require the mentioned supervision. Some approaches use an auxiliary task to define the loss between input and generated images; for instance, Bousmalis et al. [94] use image-level classification while Hoffman et al. [95] use semantic segmentation as auxiliary tasks. Other approaches focus on appearance of the input and generated images. Shrivastava et al. [96] proposed an identity loss between the input and generated images. One restriction of this approach is that the source and target domain images have similar appearances. Zhu et al. [97] and Kim et al. [98] followed the cycle idea; i.e., from source images, target-style ones are generated which, in turn, are the input to generate new source-style images. The source-to-target and the target-to-source are different generators. In each domain, we have different discriminators. The cycle idea is not only useful because it does not require image GT, but also because the input and transformed images can have a relatively different appearances, especially compared to the approach in [96]. In other words, in contrast to other GAN proposals, a GAN trained according to the cycle idea has the potential of properly transforming the appearance of source images showing content unseen during its training. Accordingly, in this paper, we follow the cycle idea. In particular, since [97] has publicly available code—called CycleGAN—we use it for the experiments in this paper.

Finally, the work with the most similar goal to that of this paper has recently been presented by Beery et al. [99]. The addressed application is animal detection and classification from static cameras. The paper evaluates the use of synthetic data for classifying animals for which it is difficult to have sufficient real-world image samples. Therefore, similarly to us, previous real-world image samples from known classes (animals) are leveraged for retraining their (animal) classifier together with the synthesized images containing the new class samples (they consider deer as a new class). In this paper, rather than focusing on one new class at a time, we also evaluate different balances between known and unknown classes. We also evaluate the difference between using the synthetic images as they come from the virtual environment in contrast to transforming them via GANs. In both cases, since our application falls into fine-grain classification, we also assess the dependency on common visual cues between seen and unseen classes.

## 3. Method

### 3.1. Overall Idea

Assume we need a classifier C such that, given an image (e.g., framing a traffic sign), it is able to assign to it a correct label from a given set K of known labels/classes (e.g., traffic sign classes). Let I be a set of images collected for training such a C. For supervised training, we need to assign one class to each image, which is usually done offline by human annotators. Let $I_{K}$ be the corresponding annotated set of images. Then, we can run a supervised machine learning algorithm that uses $I_{K}$ to generate a classifier $C_{I_{K}}$, which will be used (at testing time) to support the addressed application (e.g., on-board traffic sign recognition). The problem arises when, during the execution of such an application, we realize that there are classes of interest not included in K (e.g., after a warning from an out-of-distribution detection module also running as part of the application). Let us call U this set of new classes such that K∩U=∅. For training a new supervised classifier $C_{I_{K\cup U}}$ which takes into account all classes, we need to collect and manually annotate new images covering a sufficient number of instances for each class in U. This may be difficult to do as quickly as we could wish, since we may be facing unusual classes (i.e., for which it is difficult to find corresponding instances by just randomly roaming in the real world) and there will also be a latency due to the manual annotation of the found instances.

Accordingly, the alternative method that we want to explore relies on automatically generating synthetic images to quickly obtain sufficient annotated instances of the new classes for training a new classifier. In addition, as we have already mentioned, training visual models using pure synthetic images can lead to a performance drop when performing in the real world. In order to reduce such a domain gap, GANs are a possible solution; i.e., by directly transforming the images of the source domain (e.g., synthetic world) to have a similar appearance to those of the target domain (e.g., real world). We follow this approach in this study.

**Figure 2:**
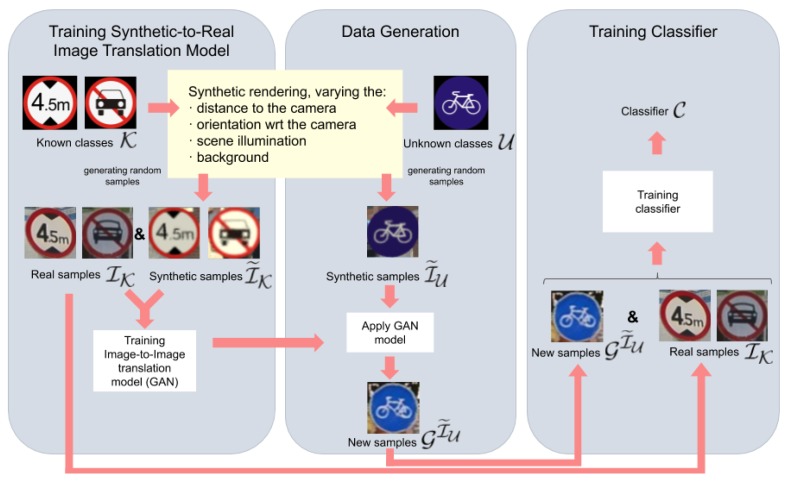
The proposed method for retraining a classifier, C, to keep detecting previously known classes (K)—for which we have labeled real-world samples—and, in addition, new previously unknown classes (U) for which we do not have real-world samples. The key idea is to have synthetic samples for both the known and new classes. The real-world samples of the known classes ($I_{K}$) and the synthetic ones (${\tilde{I}}_{K}$) are used to train a Generative Adversarial Network (GAN) with the aim of performing synthetic-to-real domain adaptation. In particular, the synthetic samples of the new classes (${\tilde{I}}_{U}$) are transformed ($G^{{\tilde{I}}_{U}}$) by this GAN. Then, the real-world samples of the previously known classes and the transformed synthetic samples of the new classes are used to train the desired classifier. The overall idea is illustrated for traffic sign recognition.

Now, let ${\tilde{I}}_{U}$ be a set of synthetically generated images automatically annotated according to the classes in U, and $G^{{\tilde{I}}_{U}}$ the corresponding set of images transformed by a GAN. We aim to train a classifier $C_{\left\{G^{{\tilde{I}}_{U}},I_{K}\right\}}$ (ideally) performing in the real world as if $G^{{\tilde{I}}_{U}}$ would consist of real-world images annotated by humans. Yet another question is how the GAN is trained. From the point of view of generating synthetic images, generating images for classes in U is analogous to generating for classes in K. Therefore, we require that besides the set of real-world images $I_{K}$, we also have a set ${\tilde{I}}_{K}$ of (automatically) annotated synthetic images for the known classes; i.e., both sets cover the same classes, but for each class, it can be a different number of samples in each set. The GAN is trained to transform images from the set ${\tilde{I}}_{K}$ into the set $I_{K}$, but without assuming a one-to-one pairing of the images from both sets. In other words, the GAN will learn to perform domain-to-domain transformations, but not class-specific transformations between domains. Therefore, when we need to transform synthetically generated instances for a new previously unknown class (i.e., in U), we can apply the previously learned GAN even if it was not exposed to such a class during the training time and, in fact, it will not be exposed at this time, due to the lack of real-world instances of this class. Figure 2 depicts the overall idea.

### 3.2. Data Generation

We start by generating synthetic images with automatic GT for each unknown class. We require a real-world example showing the appearance of an instance of each unknown class (i.e., the example already used to decide that the class must be considered in future versions of the classifier). Then, a designer can create a textured 3D model of it. This model can then be populated in a virtual environment that we have predefined. Next, we can capture as many images as we need containing instances of the new class along with automatic GT, which is done under predefined variations regarding the environmental and image capture conditions. For instance, for the traffic sign recognition study we address in this paper, we perform the following steps: (1) We create a traffic sign 3D model for a given unknown sign; (2) we use the SYNTHIA environment [44] to populate the 3D model in locations predefined for traffic signs; (3) we automatically aim the camera that captures images towards these locations, varying the capturing angle and distance between the camera and the traffic sign, as well as the scene illumination. This procedure ensures visual variability in the collected images due to the fact that environmental shadows influence the captures, as well as global illumination, resolution, etc. The same procedure can be used to capture synthetic images of known classes intended to be used in the training of the domain-adaptation GAN. In the case of the traffic signs, using the pixel-wise semantic segmentation GT provided by the virtual environment (SYNTHIA), we create corresponding 2D bounding boxes, which we crop to obtain the final synthetic image samples.

As we have mentioned before, synthetic images depicting instances of new classes must still be transformed by means of a GAN in order to alleviate domain shift effects. With this aim, we used the publicly available implementation of CycleGAN—as detailed in [97]—which we train using images of known classes taken from the synthetic and real-world domains. The adversarial loss aiming at approaching the appearance of synthetic and real-world images is defined as follows:

(1) $$L_{adv}\left(G_{A\to B},D_{B},I^{A}\right)=E_{i^{}∼I^{A}}\left[log\left(D_{B}\left(G_{A\to B}\left(i^{}\right)\right)\right)\right],\;$$

where *A* and *B* are different domains (synthetic or real in our case), $G_{A\to B}$ refers to the GAN generator from domain *A* to domain *B*, $D_{B}$ refers to the GAN discriminator that distinguishes between images really coming from domain *B* and those put out by $G_{A\to B}$, and $I^{A}$ is a set of images from domain *A*. The GAN discriminator is trained according to the following loss:

(2) $$L_{disc}\left(G_{A\to B},D_{B},I^{A},I^{B}\right)=E_{i^{B}∼I^{B}}\left[log\left(D_{B}\left(i^{B}\right)\right)\right]+E_{i^{A}∼I^{A}}\left[log\left(1-D_{B}\left(G_{A\to B}\left(i^{A}\right)\right)\right)\right].\;$$

In addition, CycleGAN uses additional losses to force the image appearance to be transformed between domains without affecting the semantic content of the transformed images. In particular, the following cyclical reconstruction loss is used:

(3) $$L_{rec}\left(G_{A\to B},G_{B\to A},I^{A}\right)=E_{i^{}∼I^{A}}\left[{∥G_{B\to A}\left(G_{A\to B}\left(i^{}\right)\right)-i^{}∥}_{1}\right],\;$$

which is complemented (regularized) with an additional loss aiming at not only ensuring in-domain content reconstruction, but also across-domain content similarity:

(4) $$L_{sim}\left(G_{A\to B},I^{A}\right)=E_{i^{}∼I^{A}}\left[{∥G_{A\to B}\left(i^{}\right)-i^{}∥}_{1}\right].\;$$

Now, we can define the total loss function to train the GAN generator that transforms images from a synthetic domain S to a real domain R as follows:

(5) $$\begin{array}{cc} L(G_{S\to R},G_{R\to S},D_{S},D_{R},I^{S},I^{R}) & =L_{adv}\left(G_{S\to R},D_{R},I^{S}\right)+L_{adv}\left(G_{R\to S},D_{S},I^{R}\right)\\ \; & +L_{disc}\left(G_{S\to R},D_{R},I^{S},I^{R}\right)+L_{disc}\left(G_{R\to S},D_{S},I^{R},I^{S}\right)\\ \; & +L_{sim}\left(G_{S\to R},I^{S}\right)+L_{sim}\left(G_{R\to S},I^{R}\right)\\ \; & +L_{rec}\left(G_{S\to R},G_{R\to S},I^{S}\right)+L_{rec}\left(G_{R\to S},G_{S\to R},I^{R}\right).\; \end{array}$$

At training time, we use $I_{K}$ and ${\tilde{I}}_{K}$ as $I^{R}$ and $I^{S}$ image sets, respectively. Then, the learned generator $G_{S\to R}$ will be the CNN that we use to transform a set of synthetic images ${\tilde{I}}_{U}$ into $G^{{\tilde{I}}_{U}}$.

## 4. Experimental Results

The experiments were designed to address two questions. Since we use synthetically generated instances of unknown classes to retrain the current classifier, we will have a domain shift problem. **(Q1)** *Can we reduce this domain shift by applying an image-to-image translation GAN to the samples of the unknown classes, provided that such a GAN was trained only with samples of the known classes?* and **(Q2)** *What are the overall classification results when training the classifier using the real-world data of the known classes with the data generated for the new classes following this GAN-based proposal?*

Note that question **Q1** focuses on classification results in terms of new classes in isolation, while **Q2** addresses the ultimate question, since we combine real-world samples from known classes with generated samples from unknown classes for training of the all-classes classifier. In the following, Section 4.1 introduces the synthetic and real-world datasets used in our experiments, and Section 4.2 elaborates the designed experiments to answer these questions, along with the obtained results and corresponding discussion.

### 4.1. Datasets

In order to perform our experiments, we need a dataset based on real-world images of traffic signs as well as another based on synthetic images. We selected the widely used Tsinghua traffic sign dataset [51] and a synthetic analog that we created to perform the research in this paper, which we call SYNTHIA-TS. We briefly describe them in the following.

**Figure 3:**
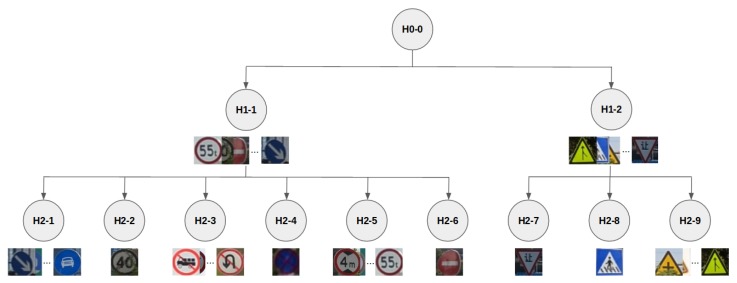
Hierarchy of Tsinghua traffic signs.

*Tsinghua* is a dataset composed of outdoor scenes captured in China while driving a car in urban scenarios. Following the approach proposed in [51], we cropped the traffic signs and removed all the classes with less than 100 samples. The resulting dataset is composed of 21,721 cropped images, representing 42 traffic sign classes. In terms of appearance, these classes can be hierarchically organized as shown in Figure 3, where the first criterion of splitting the dataset is the external shape of the traffic signs, and the second is the textual/graphical content of the signs. Both the shape and content define the semantics of each traffic sign, i.e., the class.

*SYNTHIA-TS* was created by mimicking the 42 classes considered from the Tsinghua dataset, using one textured 3D model per each of those classes. Then, following the protocol explained in Section 3.2, we acquired traffic sign images within the SYNTHIA environment. The generated data is balanced for all image acquisition conditions and classes. We generated 23,222 instances in total, covering the 42 classes. Since the SYNTHIA environment was previously created for multiple purposes, obtaining these instances from it took less than 2 h using a desktop PC based on an INTEL Core i7 CPU and one NVIDIA Geforce GTX 1080 GPU.

### 4.2. Experiments: Design, Results, and Discussion

We have not only considered the Tsinghua and SYNTHIA-TS datasets as a whole, i.e., H0-0 in terms of the hierarchy shown in Figure 3; instead, in order to perform a finer-grained analysis regarding questions **Q1** and **Q2**, we also conducted experiments based on different nodes of this hierarchy, which we call *splits*. Accordingly, our setup assumes that we have an existing split $s_{1}$ of real-world annotated images for training, and that we also want to learn a new split $s_{2}$, for which we have no access to a proper amount of corresponding real-world images and, therefore, we have to synthesize them. It is understood that $s_{1}$ and $s_{2}$ have no intersection between classes. On the other hand, for the purpose of performing comparative evaluations in our experimental setting, we do in fact have access to the real-world annotated images of split $s_{2}$.

Since we will be referring to splits coming from synthetic and real-world data, the former sometimes transformed by a GAN, and the latter sometimes used as training or testing data, we have defined the compact notation of Table 1, which will allow us to be precise and concise when describing the multiple experiments we report in this section. Using this notation and given two splits $s_{1}$ and $s_{2}$, an example of an experiment for **Q2** would consist of using $T_{s_{1}}^{T}$ and $S_{s_{2}}^{T}$ to train a traffic sign classifier for the known classes in split $s_{1}$ together with the new classes in split $s_{2}$, which we would like to be accurate when testing in $T_{s_{1}\cup s_{2}}^{C}$, i.e., accurate for all classes. Alternatively, if we use a GAN to transform the synthetic images, then the training of the classifier would be done with $T_{s_{1}}^{T}$ and ${G^{s_{2}}}_{s_{1}}$. In fact, we transformed all of the synthetic images at once, which took less than 1.5 h using a desktop PC based on an INTEL Core i7 CPU and one NVIDIA GeForce TITAN X Pascal GPU.

As we can see in Figure 3, we have three hierarchical levels: (1) The whole data, (2) two splits based on external shape, and (3) given a shape, different data based on content. Each considered split is defined in Table 2, which specifies their features. We do not consider splits with only one class (i.e., H2-2, H2-4, H2-6, H2-7, and H2-8) since they would not allow the addressing of **Q1** (for which at least two classes are needed). However, note that although these splits are not considered in isolation, their data is considered when working with a split corresponding to their parent nodes in the hierarchy.

Now, we start the experiments by establishing the upper and lower bounds of different traffic sign classifiers. In these experiments, we use the full Tsinghua and SYNTHIA-TS datasets. Therefore, in this case, we use the split H0-0 (Figure 3) for Tsinghua, i.e., we use $T_{H0-0}^{T}$ and $T_{H0-0}^{C}$ for training and testing, respectively. Both sets have samples of all of the traffic signs that we consider. More specifically, for each class, 60% of the samples are used for training tasks (CycleGAN and traffic sign classifiers) and the remaining 40% for testing traffic sign classifiers. The per-class training/testing sampling is performed randomly and once. Training on $S_{H0-0}^{T}$ and testing on $T_{H0-0}^{C}$ acts as the lower bound, since we are using only synthetic images (as they come from the virtual environment); therefore, we must expect a domain shift. Training on $T_{H0-0}^{T}$ acts as the upper bound, since we are using real-world images from the same distribution (camera and world area) as in the testing set. Table 3 shows these upper and lower bound results for the different architectures that we have considered, namely VGG16 and ResNet101. Moreover, since during the training of CNNs, there is certain amount of randomness (e.g., when sampling the datasets during a mini-batch), we repeat each training five times and report testing accuracy in terms of the mean and standard deviation of the F1 classification score (i.e., F1 = (2TP)/(2TP + FN + FP)) computed on the respective classification results. These results show that: (1) We can achieve a high classification accuracy with the appropriate real-world data; (2) using the synthetic data for training produces a reasonable accuracy (far from random), but there is a dramatic domain shift, with results dropping from 97.59% to 36.05% for VGG16, and from 98.76% to 58.74% for ResNet101.

**Figure 4:**
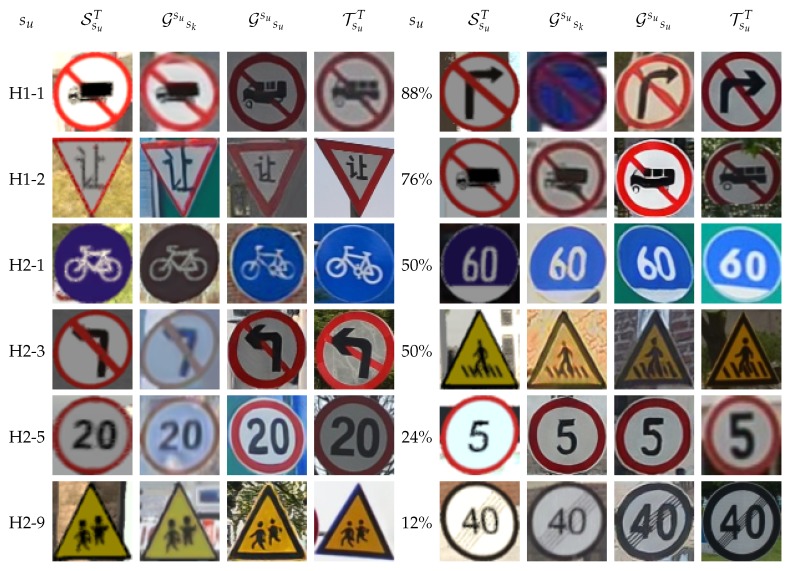
Sample images. The left block (4 columns) and right block (4 columns) rely on different criteria to generate their splits. Left block: Splits based on the hierarchy shown in Figure 3. Right block: Splits based on the balance between known and unknown (shown %) classes. Within each block, row-wise, we show samples from the same class of the unknown split. Within each block, from the left to the right column, we have: A SYNTHIA-TS sample from the unknown class, a SYNTHIA-TS sample from an unknown class transformed by a CycleGAN trained on SYNTHIA-TS-to-Tsinghua samples from known classes, as in the previous column but training a CycleGAN on the unknown classes, and a Tsinghua sample of an unknown class.

Table 4 and Table 5 report results to answer **Q1**. We consider paired splits—one is used as the set of known classes ($s_{k}$), and the other as the set of unknown classes ($s_{u}$). These splits do not intersect, but their union does not necessarily correspond to the full traffic sign hierarchy, because only splits from Table 2 are considered. The pairs were designed to force different global appearances between known and unknown classes. The $S_{s_{u}}^{T}$ columns report the lower bound of classification accuracy for each experiment, i.e., training a classifier for classes in $s_{u}$ with samples in $S_{s_{u}}^{T}$ but testing on the real-world data $T_{s_{u}}^{C}$. Columns $T_{s_{u}}^{T}$ act as the upper bound, since training is done on real-world samples of $s_{u}$ as if they were actually known. Columns ${G^{s_{u}}}_{s_{k}}$ report the classification accuracies when training is done with the samples of $G^{s_{u}}$, i.e., the samples of $S_{s_{u}}^{T}$ transformed by a CycleGAN trained to perform image-to-image translation from $S_{s_{k}}^{T}$ to $T_{s_{k}}^{T}$. Therefore, the CycleGAN has not seen samples from classes in $s_{u}$ at training time. Finally, we also include the case ${G^{s_{u}}}_{s_{u}}$, where the CycleGAN has been trained using samples from the unknown set of classes. Obviously, this is not realistic in our application setting; however, it can be taken as an upper bound of the accuracy, which would be possible to achieve by using CycleGAN to transform the synthetic images. Figure 4 shows examples of the images involved in our experiments: Synthetic, real, and transformed by different CycleGANs.

These results based on splits confirm the observations made for H0-0 according to Table 3; i.e., training and testing (for the unknown classes) with real-world data shows high classification accuracies, while training with the pure synthetic data and testing in the real-world data shows a significant drop of accuracy. Again, ResNet101 is more robust to domain shifts than VGG16. We can see how the gap gets larger as the number of classes based on synthetic data (unknown ones) increases. For instance, the gap for H1-1 is larger than for H1-2, both for VGG16 and ResNet101. Note that H1-1 contains 35 classes and H1-2 only seven (see Table 2). If we analyze the splits of the next hierarchical level (H2-X), the same observations hold; note that H2-3 and H2-5 (10 and 16 classes, respectively) show a larger gap than H2-1 and H2-9 (six and five classes, respectively), both for VGG16 and ResNet101.

**Figure 5:**
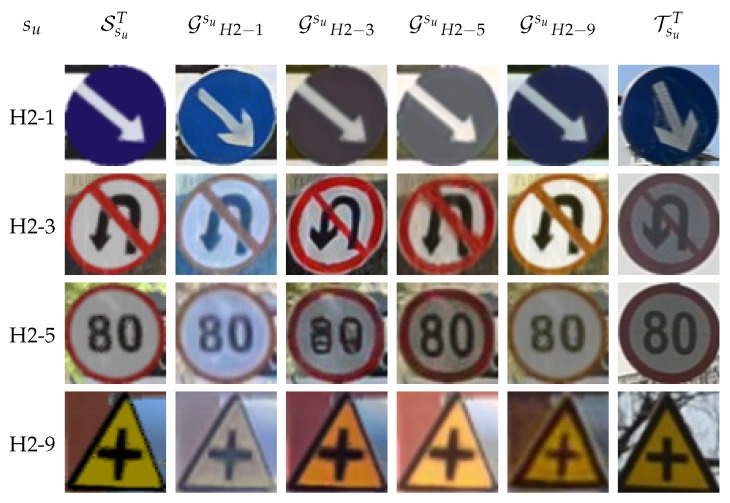
Samples based on H2-X splits (Figure 3). Rows: Splits with classes in the role of unknown. Left-to-right columns: Samples from SYNTHIA-TS, SYNTHIA-TS samples transformed by a CycleGAN trained on SYNTHIA-TS, and Tsinghua samples of classes in H2-1 split—which play the role of known classes here—, analogous for H2-3 instead of H2-1, for H2-5 and H2-9, and samples from Tsinghua.

On the other hand, CycleGAN indeed helps to significantly reduce the domain shift. When using the H1-1 split as known classes to train the CycleGAN, and applying this GAN to the synthetic images of the unknown classes—i.e., those in H1-2 split—we see ∼9 points of accuracy gain when testing in real-world images of the H1-2 split (9.21 for VGG16 and 9.60 for ResNet101). Changing the roles of these splits, the gain is 10.68 for VGG16 and 4.56 for ResNet101. However, in the two situations (H1-1/H1-2 as known/unknown and vice versa), ResNet101 reports significantly higher accuracies (more than 10 points) after the GAN-based domain adaptation of the synthetic images. In addition, for VGG16 and ResNet101, H1-2 as the split of unknown classes shows significantly higher accuracies (more than 20 points) than when it is H1-1, which is just a consequence of starting with similar accuracy differences before domain adaptation. Looking to the H2-X splits, we can see that the GAN-based domain adaptation reports significantly higher accuracy in most of the experiments. In fact, it is more interesting to analyze when it is not the case. For instance, when split H2-1 is used to train the CycleGAN, we obtain either very low accuracy gains (e.g., for VGG16, 1.43 when the unknown classes are in H2-5 and 2.19 for H2-9) or even negative adaptation (e.g., –2.93 for H2-3 with VGG16, and for H2-3/5/9 with ResNet101). We think that, when using H2-1 to train the CycleGAN, the learned image-to-image transform is too biased towards a blue background, which is a color not present in the rest of the considered H2-X splits (in the role of unknown classes). When exchanging the roles between H2-1 and the rest of the considered H2-X splits, the conclusion is the same for VGG16. However, ResNet101 is still able to extract the most from the domain-adapted images, showing significant accuracy gains with respect to using the synthetic images as they come directly from the virtual environment. Figure 5 presents some visual hints. For instance, when split H2-1 is used to train the CycleGAN, this adds a bluish color to the transformed images; when the CycleGAN is trained with the H2-9 split, the added color is yellowish. The former is more marked than the latter, which may be the reason behind some of the previously mentioned cases of poor domain adaptation. We can see other effects, like blue background images going to black backgrounds. According to the reviewed results, ResNet101 seems more robust to this effect than VGG16 (see the case of $s_{u}=H2-1$ in Table 4 and Table 5).

Table 4 and Table 5 help to analyze results in scenarios where there are significant visual differences among the known/unknown classes. We are also interested in analyzing different balances between known and unknown classes. Analogously to Table 1, we will define splits denoted by a percentage of classes; e.g., 100% would be H0-0. Each of these splits also has a complementary one with the remaining classes. When forming the new splits, in order to be sure that we do not degenerate in the previous hierarchy-based experiments, the classes are not sampled from H0-0, but they are proportionally and randomly sampled from all of the H2-X splits. For example, 50% would consider half of the H2-1, H2-3, H2-5, and H2-9 classes, added to H2-2, H2-6, and H2-8, which only have one class. Table 6 and Table 7 present the corresponding results. We can see how previous observations are confirmed, namely: (1) Domain gap increases with the number of synthetic classes (the unknown ones) to be covered by the traffic sign classifier, but still, the obtained accuracies are reasonable; (2) CycleGAN is able to dramatically reduce the domain shift for the unknown classes, recovering from ∼10 to even ∼30 points of accuracy; (3) ResNet101 is able to produce the best results before and after domain adaptation.

Overall, to already answer **Q1**, we see that using known classes to train a GAN-based transformation from synthetic to real-world domains indeed helps to dramatically reduce the classification accuracy gap due to the domain shift for synthetically generated new classes. However, there are scenarios more favorable than others, and there is still room for improvement.

First, the CNN used matters. Here, Resnet101 shows significantly better classification accuracies than those of VGG16; i.e., ResNet101 is more robust to this kind of known/unknown class setting. We can see this by looking at Table 4 and Table 5. Note how, for H2-X splits, when the split of classes used to train CycleGAN is the same split as that used to train the traffic sign classifier (${G^{s_{u}}}_{s_{u}}$ columns), then the classification accuracies of VGG16 and ResNet101 are similar, and VGG16 even outperforms ResNet101 several times. A similar effect can be appreciated in Table 6 and Table 7. Hence, ResNet101 seems to be more robust to image imperfections introduced by CycleGAN. In this favorable but unrealistic setting, the domain-adapted images show fewer artifacts (see Figure 4 and Figure 5).

Second, the most adverse scenario is indeed when known and unknown classes show very different appearances combined with a low known/unknown class ratio. In a reasonable case, as for ∼(88%/12%) splits of randomly selected known/unknown classes, using ResNet101, we can see how we obtain a classification accuracy of 97.24 on average (Table 7) where training with real-world data reaches 100.00. In the most imbalanced case, ∼(12%/88%), ResNet101 reaches a classification accuracy of 77.85, still far from the 98.82 when training with real-world images. Note that in this scenario, there is room to improve GAN-based image-to-image translation, since even using the classification classes to train the CycleGAN, the obtained accuracy is 84.93, still far from 98.82.

Although in this paper, these are just intermediate results on our way to address **Q2**, this analysis is already useful if our goal is to perform transfer learning for the traffic sign classifier; i.e., if we want to train a classifier that only needs to operate in a new set of traffic signs for which we do not have enough samples, and we want to leverage knowledge from the known classes even if these are not going to be used for classification anymore—for instance, in particular environments with specialized traffic signs, like in some closed infrastructures or industrial facilities. In fact, the vision system does not need to be onboard a vehicle; it can even be on a humanoid or any other robotic platform. However, a probable requisite in this case would be to use the same camera sensor model to classify new classes as that used for collecting the real-world images involved in the training of the GAN-based domain adapter.

Finally, in order to address **Q2**, we performed the experiments shown in Table 8 and Table 9. In these tables, columns $S_{H0-0}^{T}+T_{s_{k}}^{T}$ refer to training jointly with synthetic and real-world data. $S_{H0-0}^{T}$ is the full SYNTHIA-TS set, i.e., covering known (*k*) and unknown (*u*) classes. Therefore, we use all of the synthetic data available for the classes we want to classify. On the other hand, $T_{s_{k}}^{T}$ refers to the training set of all known real-world classes; in these experiments, k∪u are the 42 considered classes of split H0-0. Therefore, $T_{s_{k}}^{T}+T_{s_{u}}^{T}$ equals the full Tsinghua training set ($T_{H0-0}^{T}$). Columns ${G^{H0-0}}_{s_{k}}+T_{s_{k}}^{T}$ are analogous to previous ones, but in this case, rather than using the synthetic data as gathered from the virtual environment, we use it transformed by a CycleGAN. These GANs are trained only using the known classes in each experiment, i.e., in this case, $T_{s_{k}}^{T}$ and $S_{s_{k}}^{T}$. Accordingly, ${G^{H0-0}}_{s_{k}}$ is composed by ${G^{s_{k}}}_{s_{k}}$, used as pure data augmentation, as well as ${G^{s_{u}}}_{s_{k}}$, which is really needed, since we assume that we do not have real-world training samples for the split $s_{u}$.

We see that the observations done in the context of **Q1** apply here too: (1) The domain gap increases with the number of synthetic classes (the unknown ones); (2) CycleGAN is able to significantly reduce the domain shift; (3) ResNet101 performs better than VGG16 before and after domain adaptation; even when using the full Tsinghua training set, they report similar upper bounds (97.59 for VGG16, 98.76 for ResNet101). In the case of ResNet101, the best cases almost reach the upper bound: (1) When the known classes are those in split H1-1 (∼83% of classes) and the unknown in split H1-2 (∼17% of classes), we obtain 97.57, which is very close to 98.76; (2) when classes are directly selected randomly on the H2-X hierarchy level, the case of 12% of unknown classes reaches 95.54, which is also a very high accuracy; even the 24% case still reports 92.57. Moreover, in all of these cases, the accuracy of the known classes keeps over 95 and over 84 for the unknown ones (91.55 for H1-2, 88.95 for the 12%, 84.78 for the 24%). Overall, we can conclude that with ResNet101, the proposed method works well when the ratio of unknown/known classes is of ∼1/4. In order to reach the upper bound, we can investigate if we can still improve CycleGAN, but in this ∼1/4 regime, the *last mile* can probably be covered by adding a small number of real-world collected and annotated samples from the unknown classes. As the vision system keeps performing in the real-world, the samples falling in the new classes can be kept; then, we can replace the synthesized and transformed samples by these self-annotated ones in a future retraining of the classifier. In fact, these self-annotation cycles can be also a good approach for more challenging ratios of unknown/known classes; note that for the 76% of unknown classes, the results are over 70, and over 50 for the 88%.

## 5. Conclusions

There are situations where a computer vision system may need to recognize new (previously unknown) classes, but the lack of samples from such classes (i.e., raw images with annotations) may seriously delay this possibility. In this paper, we have explored how to address this situation by using synthetic data and leveraging samples from the classes already known to the system. Since there is a domain shift between synthetic and real worlds, addressing the problem involves incorporating some kind of domain adaptation. To solve the problem of the lack of data, we have proposed to teach a GAN using (the already available) samples from the known classes, and to apply it to adapt synthetic samples from the new classes. As a proof of concept, we have focused on traffic sign recognition. We have used the publicly available Tsinghua dataset and we have created a synthetic dataset (SYNTHIA-TS) for designing the experiments presented in this paper. In particular, the experiments have been designed to address two questions. First, we addressed the intermediate question *can we reduce the synthetic-to-real domain shift by applying an image-to-image translation GAN to the unknown classes, provided that such a GAN was trained only with the known classes?* After an extensive set of experiments and results that we have presented and discussed, the answer was positive, which leads us to the main question, namely, *what are the overall classification results when training the classifier using the real-world data of the known classes with the data generated for the new classes following our proposal?* Again, the obtained results allow us to conclude that the proposed method is indeed effective, provided that we use a proper CNN to perform the classifications task, as well as a proper GAN to transform the synthetic images. Here, a ResNet101-based classifier and domain adaptation based on CycleGAN performed extremely well for ratios of unknown/known classes of even ∼1/4. For more challenging ratios such as ∼4/1, the results are also very positive. As a matter of fact, instead of focusing on improving the components of the presented method, as future work, we plan to augment this method with complementary techniques such as self-annotation, i.e., using the classifier generated with our current method to self-annotate real-world samples of the new classes for a posterior retraining/fine-tuning of the classifier. The datasets of synthetic traffic signs used in this paper are publicly available at www.synthia-dataset.net.

## Figures and Tables

**Table 1:** Basic notation for data subsets.

$S_{s_{}}^{T}$	Synthetic training data from split $s_{}$
$T_{s_{}}^{T}$	Tsinghua training data in split $s_{}$
${G^{s_{2}}}_{s_{1}}$	Dataset $G^{s_{2}}$ generated by applying a GAN trained on splits $S_{s_{1}}^{T}$ & $T_{s_{1}}^{T}$ to split $S_{s_{2}}^{T}$
$T_{s_{}}^{C}$	Tsinghua testing (classification) data from split $s_{}$

**Table 2:** Splits of Figure 3 used in appearance-driven experiments. Tsinghua samples are divided as training and testing tasks (details in main text). SYNTHIA-TS samples are used in training tasks only.

Split	Num.	Samples	Samples	
Code	Classes	Tsinghua	SYN.-TS	Content
H0-0	42	21,721	23,222	All the traffic sign classes are considered
H1-1	35	20,256	19,507	Only circular traffic signs
H1-2	7	1465	3773	Only non-circular traffic signs
H2-1	6	3713	3248	Mandatory actions (circular, blue backg., white border).
H2-3	10	4012	5885	Prohibition actions (circular, white backg., red border, and diag. line)
H2-5	16	7316	8896	Information (circular, white backg., with red border)
H2-9	5	989	2713	Working areas (triangular, yellow backg., black border)

**Table 3:** Lower and upper bounds for traffic sign classification on $T_{H0-0}^{C}$. Average and standard deviation F1 scores for five training-testing runs are shown. The lower bound corresponds to training only with synthetic data ($S_{H0-0}^{T}$), while the upper bound corresponds to training with real data ($T_{H0-0}^{T}$).

Training Set	VGG16	ResNet101
$S_{H0-0}^{T}$	36.05±3.92	58.74±2.04
$T_{H0-0}^{T}$	97.59±0.15	98.76±0.14

**Table 4:** Experiments to support **Q1** (see main text). All tests are done in $T_{s_{u}}^{C}$. Average and standard deviations of F1 scores are reported, since each experiment is performed five times. The column ${G^{s_{u}}}_{s_{k}}-S_{s_{u}}^{T}$ just stands for the subtraction of the means of the respective columns.

Known	Unknown	VGG16	VGG16	VGG16	VGG16	VGG16
Classes ($s_{k}$)	Classes ($s_{u}$)	$S_{s_{u}}^{T}$	${G^{s_{u}}}_{s_{k}}$	${G^{s_{u}}}_{s_{k}}-S_{s_{u}}^{T}$	${G^{s_{u}}}_{s_{u}}$	$T_{s_{u}}^{T}$
H1-2	H1-1	39.20±2.99	49.88±3.68	10.68	74.55±2.67	97.44±0.23
H1-1	H1-2	65.44±4.39	74.65±4.71	09.21	94.35±0.73	94.23±6.11
H2-3			65.45±4.05	−04.84		
H2-5	H2-1	70.29±5.31	65.91±9.28	−04.38	90.81±0.71	99.22±0.28
H2-9			74.01±7.71	03.72		
H2-1			39.10±2.08	−02.93		
H2-5	H2-3	42.03±4.44	62.87±4.11	20.84	88.15±2.37	97.29±0.44
H2-9			55.92±0.72	13.89		
H2-1			54.51±1.33	01.43		
H2-3	H2-5	53.08±7.75	60.85±4.90	07.77	85.25±1.48	97.35±0.25
H2-9			61.59±2.88	08.51		
H2-1			72.51±5.98	02.19		
H2-3	H2-9	70.32±5.90	80.35±4.09	10.03	94.77±1.03	91.81±7.60
H2-5			76.82±4.12	06.50		

**Table 5:** Experiments to support **Q1** analogously to Table 4, but using ResNet101.

Known	Unknown	ResNet101	ResNet101	ResNet101	ResNet101	ResNet101
Classes ($s_{k}$)	Classes ($s_{u}$)	$S_{s_{u}}^{T}$	${G^{s_{u}}}_{s_{k}}$	${G^{s_{u}}}_{s_{k}}-S_{s_{u}}^{T}$	${G^{s_{u}}}_{s_{u}}$	$T_{s_{u}}^{T}$
H1-2	H1-1	58.44±1.92	63.00±2.15	04.56	84.35±1.08	98.70±0.24
H1-1	H1-2	79.20±4.06	88.80±0.52	09.60	92.06±1.66	96.26±0.83
H2-3			76.66±1.59	10.27		
H2-5	H2-1	66.39±2.05	72.65±2.90	06.26	89.71±0.88	99.42±0.13
H2-9			78.47±3.26	12.08		
H2-1			48.12±4.61	−07.01		
H2-5	H2-3	55.13±2.81	68.86±2.83	13.73	87.99±2.86	97.84±0.42
H2-9			52.70±2.82	−02.43		
H2-1			58.73±3.16	−02.49		
H2-3	H2-5	61.22±1.87	63.61±3.37	02.39	82.52±0.76	97.08±0.26
H2-9			65.11±2.09	03.89		
H2-1			67.62±5.63	−02.83		
H2-3	H2-9	70.45±4.78	82.05±2.29	11.60	92.83±0.91	90.18±0.91
H2-5			83.42±2.10	12.97		

**Table 6:** Experiments to support **Q1** (see main text). All tests are done in $T_{s_{u}}^{C}$. Average and standard deviations of F1 scores are reported, since each experiment is performed five times. The column ${G^{s_{u}}}_{s_{k}}-S_{s_{u}}^{T}$ just stands for the subtraction of the means of the respective columns.

Known ($s_{k}$)/Unknown ($s_{u}$)	VGG16	VGG16	VGG16	VGG16	VGG16
% Classes; Num. Classes	$S_{s_{u}}^{T}$	${G^{s_{u}}}_{s_{k}}$	${G^{s_{u}}}_{s_{k}}-S_{s_{u}}^{T}$	${G^{s_{u}}}_{s_{u}}$	$T_{s_{u}}^{T}$
~88%/12% ; 37/05	82.58±3.62	91.84±0.92	09.26	99.08±0.42	99.85±0.18
~76%/24% ; 32/10	78.72±1.57	88.74±0.86	10.02	95.00±0.54	99.30±0.20
~50%/50% ; 20/21	41.80±2.85	74.11±1.36	32.31	85.85±1.15	97.75±0.46
~24%/76% ; 10/32	43.81±2.22	71.31±2.64	27.50	82.62±2.47	97.53±0.30
~12%/88% ; 05/37	40.35±2.69	50.24±1.25	09.89	76.26±3.63	94.93±0.34

**Table 7:** Experiments to support **Q1** analogously to Table 6, but using ResNet101.

Known ($s_{k}$)/Unknown ($s_{u}$)	ResNet101	ResNet101	ResNet101	ResNet101	ResNet101
% Classes; Num. Classes	$S_{s_{u}}^{T}$	${G^{s_{u}}}_{s_{k}}$	${G^{s_{u}}}_{s_{k}}-S_{s_{u}}^{T}$	${G^{s_{u}}}_{s_{u}}$	$T_{s_{u}}^{T}$
~88%/12% ; 37/05	97.54±0.69	97.24±2.27	09.70	99.47±0.46	100.00±0.00
~76%/24% ; 32/10	78.33±3.08	88.12±1.33	09.79	95.02±0.84	99.69±0.14
~50%/50% ; 20/21	63.78±1.66	78.84±2.24	15.06	87.11±1.20	98.46±0.24
~24%/76% ; 10/32	60.39±3.27	77.68±1.85	17.29	86.34±1.05	98.75±0.13
~12%/88% ; 05/37	60.17±2.61	59.12±2.61	−01.05	84.42±0.76	96.22±0.06

**Table 8:** Experiments to support **Q2** (see main text), all done in $T_{H0-0}^{C}$. Average and standard deviations of F1 scores are reported, since each experiment is performed five times. This is done for the all-classes classification problem, but we also show detailed results for known and unknown classes. Table 3 shows the lower and upper bounds for these experiments, i.e., training only on either SYNTHIA-TS or Tsinghua data. In terms of average F1, these bounds are 36.05 and 97.59, respectively.

Unknown	VGG16, $S_{H0-0}^{T}+T_{s_{k}}^{T}$	VGG16, ${G^{H0-0}}_{s_{k}}+T_{s_{k}}^{T}$
Classes ($s_{u}$)	H0-0 ($s_{k}$/$s_{u}$)	H0-0 ($s_{k}$/$s_{u}$)
H1-1	37.12±2.47(59.83±8.55/32.58±2.90)	59.54±3.60(84.97±2.89/54.45±3.82)
H1-2	88.81±1.70(95.92±0.74/53.22±9.91)	92.88±1.42(96.77±0.62/73.46±6.21)
12%	87.53±1.55(92.50±0.81/50.69±7.88)	93.79±1.16(94.73±1.16/86.83±2.11)
24%	78.17±1.81(88.21±0.68/46.05±5.79)	89.91±2.19(92.84±1.17/80.53±5.79)
50%	56.52±3.84(75.85±1.50/35.26±6.60)	74.96±1.40(85.01±1.26/63.90±2.73)
76%	30.69±2.13(49.09±1.67/24.94±3.10)	67.45±1.41(74.04±2.71/65.39±1.28)
88%	31.46±3.18(33.34±3.66/31.23±3.27)	53.62±2.26(64.44±4.74/50.70±2.46)

**Table 9:** Experiments to support **Q2** analogously to Table 8, but using ResNet101. In this case, the lower and upper bounds are 58.74 and 98.76, respectively.

Unknown	ResNet101, $S_{H0-0}^{T}+T_{s_{k}}^{T}$	ResNet101, ${G^{H0-0}}_{s_{k}}+T_{s_{k}}^{T}$
Classes ($s_{u}$)	H0-0 ($s_{k}$/$s_{u}$)	H0-0 ($s_{k}$/$s_{u}$)
H1-1	65.67±0.66(95.42±0.80/59.72±0.68)	70.65±1.60(98.53±0.38/65.08±1.97)
H1-2	95.55±0.39(98.37±0.27/81.45±3.30)	97.57±0.31(98.77±0.11/91.55±1.33)
12%	90.05±1.67(94.11±0.62/60.00±9.71)	95.54±0.82(96.44±0.47/88.95±3.49)
24%	82.70±1.22(91.02±0.63/56.07±3.15)	92.57±0.36(95.00±0.50/84.78±0.77)
50%	68.03±2.03(81.92±0.95/52.74±3.32)	81.08±0.57(87.72±0.60/73.78±0.85)
76%	49.23±1.17(50.94±1.66/48.70±1.06)	71.94±1.76(71.50±2.38/72.08±1.75)
88%	52.95±2.31(43.77±1.99/54.06±2.53)	56.56±2.56(56.83±1.93/55.43±2.70)
